# A novel model of appendicitis and appendectomy to investigate inflammatory bowel disease pathogenesis and remediation

**DOI:** 10.1186/1480-9222-16-10

**Published:** 2014-06-13

**Authors:** Rajkumar Cheluvappa

**Affiliations:** 1 Department of Medicine, St George Clinical School, University of New South Wales, Sydney, NSW, Australia; 2 Inflammation and Infection Research Centre, School of Medical Sciences, Wallace Wurth Building, University of New South Wales, Gate 9 High Street, Sydney, NSW 2052, Australia

**Keywords:** Appendicitis, Appendectomy, Inflammatory bowel disease, Colitis, Autophagy, Antigen-processing

## Abstract

The appendix contains copious lymphoid tissue and is constantly exposed to gut flora. Appendicitis and appendectomy (AA) has been shown to prevent or significantly ameliorate ulcerative colitis. In our novel murine AA model, the only existing experimental model of AA, the appendiceal pathology closely resembles that of human appendicitis; and AA offers an age-, bacteria- and antigen-dependent protection against colitis. Appendicitis and appendectomy performed ***in the most proximal colon*** curbs T helper 17 cell activity, curtails autophagy, modulates interferon activity-associated molecules, and suppresses endothelin vasoactivity-mediated immunopathology/vascular remodelling ***in the most distal colon***. These AA-induced changes contribute to the limitation/amelioration of colitis pathology. Investigating strategies to manipulate and modulate different aspects of these pathways (using monoclonal antibodies, combinatorial peptides, and small molecules) would offer novel insight into inflammatory bowel disease pathogenesis, and will augment the development of new therapeutic options to manage recalcitrant colitis.

## Appendicitis-appendectomy and inflammatory bowel diseases

The vermiform appendix or simply the appendix, contains copious lymphoid tissue and is constantly exposed to gastrointestinal bacteria. Appendicitis is the most common abdominal emergency requiring surgery [1], its highest occurrence being between 10 and 30 years [2]. As succinctly summarised and critically appraised/re-analysed by Koutroubakis et al. [3,4], and in over a dozen individual clinical studies [4-17], Appendicitis and appendectomy (AA) has been shown to prevent or significantly ameliorate ulcerative colitis. This intriguing phenomenon was demonstrable only in patients who had undergone appendectomy prior to 20 years of age. AA has also shown to be protective against Crohn’s disease in a few studies [16,18]. However, other studies [11,19-21] have showed a slightly elevated risk early after appendectomy, but this has been clearly attributed to diagnostic bias (misdiagnosis of early Crohn’s disease as appendicitis) by these studies.

## Procedure overview and rationale minutiae - mouse model of appendicitis and appendectomy, and tissue processing

**Figure 1:**
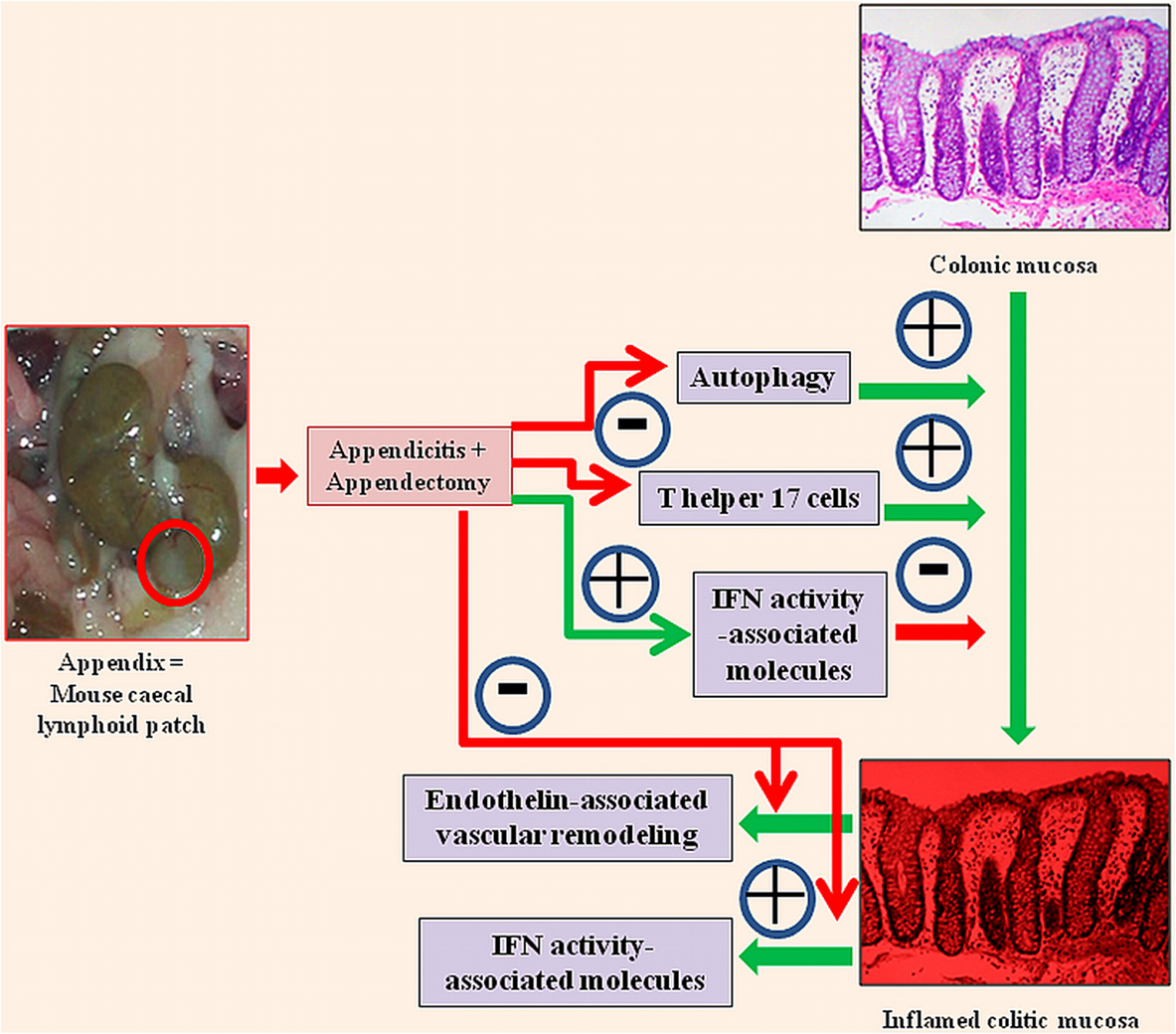
**Biological markers of inflammation and immunity correlating with our murine model of appendicitis (and appendectomy) **[24]**,**[25]**.** Appendicitis and appendectomy performed ***in the most proximal colon*** induce gene expression changes to curb T helper 17 cell activities, curtail autophagy, modulate interferon activity-associated molecules, and suppress endothelin vasoactivity-mediated immunopathology/vascular remodelling ***in the most distal colon***. These contribute to the limitation/amelioration of colitis pathology.

(1) In mice, the major caecal lymphoid patch is the equivalent of the human appendix (Figure 1). In our AA model, the appendiceal pathology closely resembles that of human appendicitis; and AA offeres an age-, bacteria- and antigen-dependent protection against TNBS-colitis [22].

(2) Balb/c mice (Male, 5 weeks), are intraperitonealy anaesthetised with xylazine (5mg/kg; Sigma-Aldrich, X1251) and ketamine (100 mg/kg; Sigma-Aldrich, K1884) followed by allocation into 2 treatment groups, the appendicitis group or the sham surgery group [23].

• Other permutations (appendicitis only or appendectomy only) do not ameliorate colitis pathology, and hence are excluded.

• Mice are randomised to have either appendicitis or sham operation.

(3) Appendicitis is induced by constructing an appendiceal pouch from the caecal lymphoid patch, which is the equivalent of the human appendix.••This appendiceal pouch (Figure 1) is obstructed by rubber band ligation using standardised negative aspiration.

(4) Sham surgery entails a similar procedure, but without continuous obstruction by band ligation of the caecal patch.

• It also involves the placement of a sterile rubber band in the abdominal cavity as a control for foreign body reaction.

• While most human ulcerative colitis patients would not have undergone any form of abdominal surgery at diagnosis, it is essential that we control for the colonic and peritoneal milieu changes induced by a simple laparotomy in our experimental AA mice group.

• Additionally, the small rubber ring used to encircle the main caecal follicle in the AA group needs to be controlled for too. Hence, in each SS group mouse, a rubber ring is introduced in the peritoneal cavity via a small laparotomy incision.

(5) Seven days following initial surgery, appendicitis mice undergo appendectomy (appendicitis and appendectomy, AA, group) by ligation and excision, while sham mice undergo a second sham surgery (sham and sham, SS, group).

(6) All mice are monitored daily.

(7) Distal colons are harvested (3 days or 14 days or 28 days post-AA) without the recta - a 1 cm-segment (from the anus) are excluded prior to harvesting the distal colon.

(8) Transmural distal colonic segments are chosen to minimise artifactual changes and maximise pathophysiological relevance; as not only superficial mucosal tissue, but also colonic lymphoid nodules/aggregations and other tissue may “transmit” the protective immunological changes induced by appendicitis and appendectomy to the distal colon.

• The proximal colon would be much more likely to be affected by the inflammatory changes experimentally induced by AA, owing to the close anatomical proximity of the main mouse caecal patch (≈human appendix) to the proximal colon.

• The novelty of our study is that the most distal regions of the large gut sustain major persistent immunological changes (protective against colitis), by manipulation at the caecum, the most proximal region of the large gut.

(9) The processing of transmural distal colonic samples consists of flushing out of faecal contents with normal saline and immediately transfer to TRIzol® reagent (50-75 mg of tissue in 600 μL of TRIzol® reagent; Invitrogen Australia Pty Limited**,** 15596-026), snap-freezing in liquid nitrogen, and storing at -80°C until the microarray analysis.

(10) After chloroform and isopropanol treatment and centrifugation followed by washing the resultant pellet with 75% ethanol, air-drying and final re-constitution in nuclease-free H_2_O is done.

(11) Concentration and purity of RNA is determined by automated optical density evaluation (OD 260/OD 280 ≥ 1.8 and OD 260/OD 230 ≥ 1.8) using Nanodrop ND-1000 (Nanodrop Technologies, Wilmington, DE, USA).

(12) The degree of RNA degradation is analyzed by the Agilent electrophoresis bioanalyzer 2100 (Agilent Technologies Inc, Santa Clara, CA, USA) with the RNA integrity number (RIN) values consistently above 7.

## Protocol for mouse model of appendicitis induction – procedure in steps

### Apparatus required

(1) Fine forceps and scissors

(2) Autoclaved bands (rings) and transfer pipettes

(3) Suture holder and sutures (Needle Eithlon 4-0)

(4) Syringes and needles (22G)

(5) Surgical platform and sterile gloves

(6) Sterilised distilled water, Falcon tubes, and tube holder frame

(7) P1000 pipettes and corresponding sterilized tips (with prior tailor-made bevelling)

(8) Sterile dressing packs (plastic sheets; plastic blue forceps, tray, sterile gauze)

(9) Shaving apparatus with shaving cream

### Reagents required

(1) 50 ml of sterile Phosphate Buffered Saline (PBS)

(2) Sterile saline (Sodium Chloride Injections BP 0.9% tubes)

(3) 5 ml anaesthetic stock solution composed of Ketamine/xlyazine/sterile PBS - A standard 5 ml anaesthetic stock solution would contain Ketamine (100mg/ml) 1 ml, Xylazine (20mg/ml) 0.25 ml, and Sterile PBS 3.75 ml.

• **Ketamine (100 mg/ml)** - 1ml for 100 mg/kg

• **Xylazine (20 mg/ml)** - 0.17 ml for 3.4 mg/kg OR 0.25 ml for 5 mg/kg

• **Sterile PBS** - 3.83 ml for 3.4mg/kg Xylazine OR 3.75 ml for 5 mg/kg Xylazine

(4) Spray bottle with 70% Ethanol

(5) Povidone-iodine

## Procedure steps

(1) Shave a circumscribed area of each mouse’s abdomen (left side) a day before surgery.

(2) Weigh each mouse and make identification marks on each mouse’s tail a day before surgery.

(3) Wrap each P1000 pipette with sterile plastic sheet (from dressing pack).

(4) Anaesthetise mouse with ketamine/xylazine/sterile PBS – Intraperitoneal injection of 0.1 ml of anaesthetic stock solution per 20 g mouse weight.

(5) Prepare surgical area observing sterile conditions meticulously:

• Use sterile gloves throughout.

• Cover bench with sterile plastic sheet and tape the corners on the hood bench.

• Use sterile plastic forceps to pick up the apparatus from the pack and put aside.

• Load autoclaved equipment onto sterile plastic sheet by the “no touching” method.

• Load bands, and pour Povidone-iodine onto sterile tray.

(6) Load band onto bevelled pipette tip with fine forceps and set aside.

(7) Clean surgical platform with ethanol and place on bench.

(8) Fix mouse to the platform by taping their arms/legs with taps.

(9) Moist eyes with a drop of saline to prevent xerosis and keratitis.

(10) Wipe abdomen with ethanol and clean the left part of the shaved abdominal wall with Povidone-iodine.

(11) Cover mouse with the sterile draper from dressing pack and make a hole in the draper above surgical area with fine forceps.

(12) Perform a ~1cm laparotomy on the left sided abdomen, using fine forceps to pick up skin and peritoneum.

(13) Mobilise caecum out to the surface with blunt curved forceps.

(14) Frequent moistening of the caecum with PBS is essential.

(15) Identify the 2 to 4 white appendiceal lymphoid patches, with one being far bigger than the others – this would be the one to do the procedure on.

(16) Attach bevelled pipette tip with band to pipette, and aspirate the lymphoid patch with firm suction pressure in gentle, but firm circular motion. Maintain suction pressure for 10 seconds before slowly unloading the band (with fine forceps) onto the neck of the newly created pouch.

• Include some colonic tissue during suction because the created pouch will get smaller as each mouse ages.

(17) Using a curved forceps carefully return bowel into abdominal cavity by gently grasping the band.

(18) Dispense 1ml of PBS in the abdominal cavity to moisten the peritoneum.

(19) Suture the abdominal wall and the skin in layers.

(20) Give 1 ml normal saline subcutaneously (scruff of neck) after the procedure.

(21) Place mice on their side and put them close to each other in the cage to prevent hypothermia.

(22) Check post-op recovery in 1 hour for complete recovery. Check recovery for 1 week post operation and record observations in post operative period table.

(23) Appendectomy is performed by 7 days after appendicitis induction by gentle separation of adhesions, ligation, and excision of the inflamed area distal to the ligature.

(24) The mice are sacrificed 3 days or 14 days or 28 days after that for distal colon harvesting.

## Data produced by this novel mouse model of appendicitis and appendectomy - results and discussion

Distal colonic gene expression studies; 3 days after surgery, or 28 days after surgery; reveal the various genes and gene-sets that are responsible for the durable protective effect of AA against colitis [23]. The gene expression data obtained from our study get more distinct between the time-points (days 3, and 28), and stabilise around day 28. Although observable in our overall microarray results, these are visually appraised better in the RT-PCR time-curves (days 3, 14, and 28) of selected genes [23]. The persistence of these expression changes would be theoretically easier to demonstrate in humans than mice, as mice have an accelerated lifespan, with 1 mouse year “often equated” to 30 human years. The 28-day-extension of our studies is adequate to demonstrate the chronological sustainability of these changes.

Using this model, we have shown that AA performed ***in the most proximal colon*** induces the following gene expression changes ***in the most distal colon*** (Figure 1):

(1) Substantial curbing of T helper 17 cell -recruitment, -differentiation, -activation, and –effector (interleukin) expression contributing to suppression of Th17 pathway-mediated immunopathologial damage [24] in colitis.

(2) Significantly and globally curbing autophagy gene expression [25], contributing to suppression of autophagy-mediated immunopathology in colitis.

(3) *Late but significant* suppression of genes and gene-sets pertaining to endothelin activity - This would suppress endothelin vasoactivity-mediated immunopathologial damage and vascular remodelling in inflammatory colitis (Manuscript submitted).

(4) No changes in interferons; but upregulation of genes pertaining to specific IFN activity-associated molecules, and downregulation of genes pertaining to other IFN activity-associated molecules (Manuscript submitted).

Using our unique murine AA model, elucidating the pathways involved in these changes will enhance the development of approaches and techniques to manipulate different genes, enzymes, and proteins related to these pathways; towards improving therapeutic options in IBD. Investigating strategies, involving monoclonal antibodies, combinatorial peptides, and small molecules (identified by high throughput screening) to manipulate and modulate different aspects of these pathways, would augment the development of new therapeutic options to manage IBD.

## Abbreviations

AA: Appendicitis-appendectomy; SS: Sham-sham; IBD: Inflammatory bowel disease; TNBS: Trinitrobenzene sulfonic acid.

## Competing interests

The author declares that he has no competing interests.
